# Automated genome mining for natural products

**DOI:** 10.1186/1471-2105-10-185

**Published:** 2009-06-16

**Authors:** Michael HT Li, Peter MU Ung, James Zajkowski, Sylvie Garneau-Tsodikova, David H Sherman

**Affiliations:** 1 Life Sciences Institute, University of Michigan, Ann Arbor, MI, USA; 2 Department of Medicinal Chemistry, University of Michigan, Ann Arbor, MI, USA; 3 Departments of Chemistry, and Microbiology & Immunology, University of Michigan, Ann Arbor, MI, USA

## Abstract

**Background:**

Discovery of new medicinal agents from natural sources has largely been an adventitious process based on screening of plant and microbial extracts combined with bioassay-guided identification and natural product structure elucidation. Increasingly rapid and more cost-effective genome sequencing technologies coupled with advanced computational power have converged to transform this trend toward a more rational and predictive pursuit.

**Results:**

We have developed a rapid method of scanning genome sequences for multiple polyketide, nonribosomal peptide, and mixed combination natural products with output in a text format that can be readily converted to two and three dimensional structures using conventional software. Our open-source and web-based program can assemble various small molecules composed of twenty standard amino acids and twenty two other chain-elongation intermediates used in nonribosomal peptide systems, and four acyl-CoA extender units incorporated into polyketides by reading a hidden Markov model of DNA. This process evaluates and selects the substrate specificities along the assembly line of nonribosomal synthetases and modular polyketide synthases.

**Conclusion:**

Using this approach we have predicted the structures of natural products from a diverse range of bacteria based on a limited number of signature sequences. In accelerating direct DNA to metabolomic analysis, this method bridges the interface between chemists and biologists and enables rapid scanning for compounds with potential therapeutic value.

## Background

Many of the pharmaceuticals currently in clinical use have natural product origins, illustrating the effectiveness of compounds found in nature as antibiotic, anticancer, cholesterol-lowering, antiparasitic and anti-fungal agents [1]. Testing for therapeutic activities remains conceptually similar compared to the time of the discovery of penicillin by Alexander Fleming. Scientists today still approach identification of medicinal compounds by screening chemicals for their ability to inhibit cellular growth. This was a landmark step for science compared to using medicines passed down through collective human experience, but one from which we have not yet significantly advanced [2].

The arrival of a powerful graphic user interface (GUI) in the mid 1990s coincided with the first whole genome sequence of the microorganism *Haemophilus influenza *[3]. This synergy of computational technology and molecular biology was the beginning of whole genome analysis aiding drug discovery by searching for robust protein targets. Genome scanning was subsequently recognized as a means of discovering useful secondary metabolites such as nonribosomal peptides (NRPs), polyketides (PKs), and terpenoids [4].

In analysis of secondary metabolism, computing abilities such as multiple sequence alignment and crystallographic analysis tools enable researchers to elucidate the functions and characteristics of key enzymes involved in assembly and tailoring of natural products including nonribosomal peptide synthetases (NRPSs) and polyketide synthases (PKSs). NRPs and PKs have long been utilized by bacteria for cellular defense and offense against competitors [5]. They are prolific in nature and generate many of the antibiotics used clinically such as penicillin, erythromycin, vancomycin, and daptomycin, among numerous others [6]. Their ability to target important biomolecules highlights their significance in the central scheme of cellular metabolism.

PKSs select simple acyl-CoAs and other extender units with an acyltransferase (AT) domain, catalyze linkage of the activated acyl chain onto a thiolation (T) domain, and decarboxylate and condense the extender units using a ketosynthase (KS) domain. Similarly, NRPSs bind and activate amino acids and other carboxylic acids through adenylation (A) and thiolation (T) domains and link them together with a condensation (C) domain to create a linear proteinogenic molecule that is either cleaved to release a linear or cyclized molecule through the action of a thioesterase (TE) [7], or in a few other cases, a reductase (RE) domain [8]. Each KS, AT, and T domain or C, A, and T domain for the PKSs and NRPSs, respectively, represent the core of one module. Most of these systems have multiple modules that each catalyzes the binding and activation of an acyl-CoA or amino acid extender unit [9].

A and AT domains are considered the gatekeepers of monomer selection of the starter and extender units that are essential for NRP and PK biosynthesis. Signature amino acid motifs determine which A or AT domain are likely to activate a particular monomer, similar in concept to how key residues, termed anticodons, in tRNAs determine which cognate amino acid it binds [10]. This stored specificity from signature residues in a linear protein sequence may be considered a hidden Markov model (HMM) [11]. Much like how multiple tRNAs may bind to the same amino acid, the NRPS A and PKS AT domain amino acid binding sequence is degenerate, such that multiple signature sequences can code for binding of the NRPS or PKS domain to the same amino acid or polyketide subunit, respectively. Multiple modifying domains including those for methylation, oxidation, epimerization, and reduction may sculpt the molecule into a highly functionalized structure. A NRPS, PKS, or hybrid NRPS-PKS enzymatic assembly-line can generate an immense chemical library that is multiplied further by enzymes within the natural product cluster to structurally modify a NRP, PK, or mixed NRP-PK molecule after its assembly through reactions such as hydroxylation, glycosylation, cyclization, and halogenation [12].

**Figure 1:**
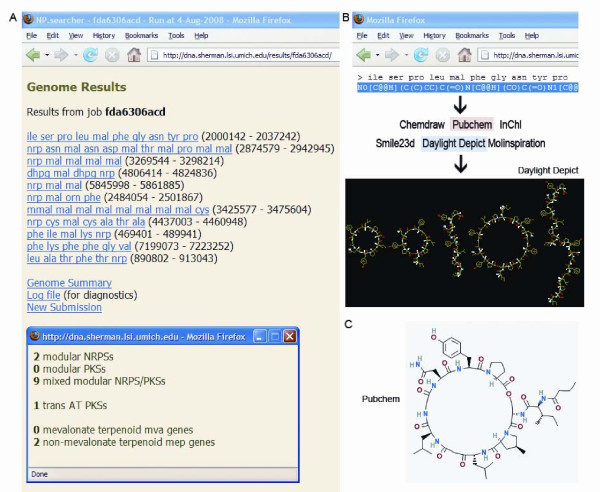
**The NP.searcher website**. Upon the uploading of a genome, NP.searcher displays the predicted NRPS/PKS gene clusters and provides a summary of natural product systems found including those comprised of trans-AT as well as terpenoid genes. (B) Clicking on any of the clusters leads to SMILES of predicted molecules produced from the biosynthetic enzymes that can be transferred into chemical software such as Daylight Depict, Molinspiration, InChI, Smile23d, and ChemDraw. (C) Individual SMILES can be input into PubChem to search for existing molecules.

The development of common letter coding for molecules, namely the Simplified Molecular Input Line Entry Specification (SMILES), has aided in organizing chemical structures within various vast chemical databases such as Zinc, NCBI, and PubChem [13]. In an effort to access the power of both genetic and chemical systems, we have developed a framework to link NCBI and other genomic repositories with chemical databases. Several programs have been developed to recognize the signature sequence of NRPS and PKS activating domains that enable limited genome mining [14,15]. A recently published software program named ClustScan produces SMILES from scanning NRPS/PKS clusters. ClustScan, however, predicts only the basic linear and cyclical conformations of a NRPS/PKS natural product [16]. The new program described in this report, NP.searcher, identifies and decodes DNA of potential natural product clusters and outputs multiple predictions for structural assembly encoded by a PKS, NRPS, or hybrid NRPS-PKS cluster, providing within seconds SMILES relating to the secondary metabolic products of a genome. NP.searcher is complementary in this aspect to ClustScan as it focuses on the combinatorial prediction of multiple unique structures that may emerge from a cluster. ClustScan is focused primarily on PKS prediction and stereochemistry of PK products. In contrast, NP.searcher was originally developed to predict NRPs and further expanded to predict the outcome of PKS systems. Furthermore, NP.searcher is open-source and can be freely modified through additional programming. We expect and encourage additional PKS and NRPS modules to be incorporated in a collaborative community environment to expand the scope and depth of this resource. NP.searcher provides public access to 2D and 3D molecular structures directly from DNA following input of nucleotide sequence information and use of the resulting SMILES. The user directly uploads the genome in FASTA format, selects the predicted natural product cluster of interest, and transfers the SMILES into any of a growing number of chemical software programs (Figure 1). The chemical structure output may also be useful in downstream molecular property and bioactivity prediction.

We tested the program using natural product biosynthetic pathways with accessible DNA sequences from two well-established databases: ASMPKS [15] for PKs and NORINE [17] for primarily NRPs and mixed NRP/PK derived systems. To enhance our natural products search engine, we also incorporated detection, though not structure generation, for trans-AT PKS systems as well as for terpenoids, that represent new niches where many more useful natural products are likely to be discovered [18].

## Implementation

### Recognizing substrate specificity

**Figure 2:**
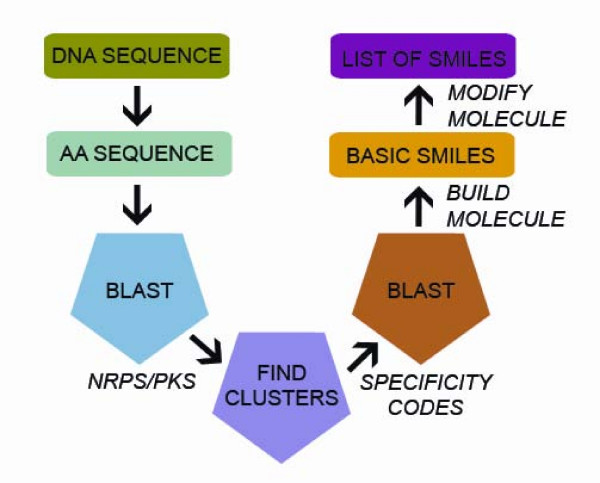
**Conversion process from DNA to SMILES**. DNA is converted to amino acid sequences, which is compared using BLAST to find clusters of catalytic domains from secondary metabolic pathways. These domains are further analyzed to find the signature sequences, or specificity codes. These codes are compared using BLAST with an internal database of signature sequences to determine the individual polyketide or peptide activated by each catalytic domain. The individual SMILES of these polyketides or peptides are concatenated to construct the basic linear molecule as a SMILES string. Additional SMILES are produced from modifications to this basic SMILES set.

**Figure 3:**
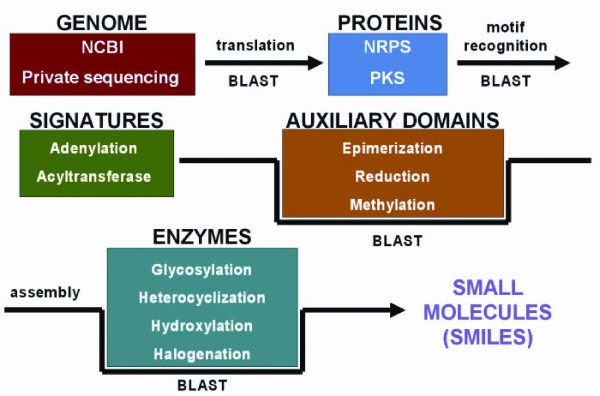
**Constructing and tailoring natural products**. Genome sequence data is translated and the NP.searcher uses BLAST to find NRPS and PKS gene clusters. BLAST is further used to find motifs of the adenylation and acyltransferase domains as well as auxiliary domains that function during chain assembly to build the core molecule. BLAST is also used to identify enzymes involved in tailoring the molecule following core molecule assembly.

The general overview of deciphering small molecules from genomes is outlined below (Figure 2). Genomic data in the form of DNA open reading frame text is translated into amino acid sequences. PKS and NRPS gene clusters are identified by searching for acyltransferase and adenylation domains, respectively. Once these clusters are identified, NP.searcher builds the molecule based on stored specificity codes representing individual peptide or polyketide subunits. This initial linear molecule is then modified to produce a list of structures that may arise from intramolecular reactions or tailoring of the metabolite by additional enzymes found in the cluster. NP.searcher distinguishes separate clusters by determining if the end of one activating domain is separated by more than 15 kilobases from the beginning of another. Separate clusters that specify three or more extension cycles are automatically stored. The cluster can then be read to output SMILES. NP.searcher uses Basic Local Alignment and Search Tool (BLAST) to align sequences with standard NRPS and PKS sequences and determine the signature residues that predict substrate specificity [19]. It also uses BLAST to recognize auxiliary domains embedded within NRPS and PKS modules such as epimerization, reduction, and methylation domains and enzymes such as cytochrome P450s (Figure 3). Although others have used HMM-specialized software to determine specificity, some straightforward programming for automated analysis of the BLAST output produced an effective method of extracting the signature residues and finding the predicted substrate specificity.

NP.searcher runs BLAST once to match a generic NRPS/PKS against an unknown NRPS/PKS. This enables alignment and extraction of the key residues of the unknown NRPS or PKS. The program runs BLAST again to align the discovered signatures with stored known signature sequences to determine substrate specificity. This is a fast, direct, and effective method of implementing BLAST to determine substrate specificity without resorting to any HMM-specific software. NP.searcher recognizes core and auxiliary NRPS and PKS domains if they align with an expectation value less than 0.1 and an identity greater than 120. Once an acyltransferase or adenylation domain of sufficient identity and length is recognized, the program parses its sequence for the signature residues as identified previously for the NRPS and additional ones found by compilation of various PKS sequences. We used a database of 187 NRPS signature sequences of ten residues and compiled PKS signature residues to build 18 PKS signature sequences of 18 residues from various published reports [20-25]. The program compares the extracted sequences and the database to reveal the substrate specificity of the catalytic domains. If a particular signature sequence is ambiguous regarding substrate selection, the program employs an auxiliary prediction system using an additive scoring algorithm to identify the most likely substrate. If this fails because of inconclusive scoring, then the NRPS or PKS residue is designated unknown and can be seen as a generic peptide or polyketide subunit in the structure.

### Constructing the natural product

NP.searcher was written in the software language C++, and modeled the assembly of NRPs and PKs as a linked list. Upon recognition of the substrate specificity of a catalytic domain, the SMILES string of the predicted chain elongation intermediate is appended to the SMILES string of the previous intermediate. During the elongation process, polyketide or peptide monomers may be modified as they are incorporated into the growing chain. Once the construction of the core NRP or PK is completed, the small molecule is modified based on a reaction list, where cyclization and modifications specified by the user or directly by program-recognized auxiliary enzymes encoded within the natural product cluster are applied. The program computes every possible molecule given the number of modifiable sites and the reactions designated. All possible products computed by the program are added to a product list, where they may be filtered by molecular mass and included in the data output.

## Results

### The breadth of recognition

**Figure 4:**
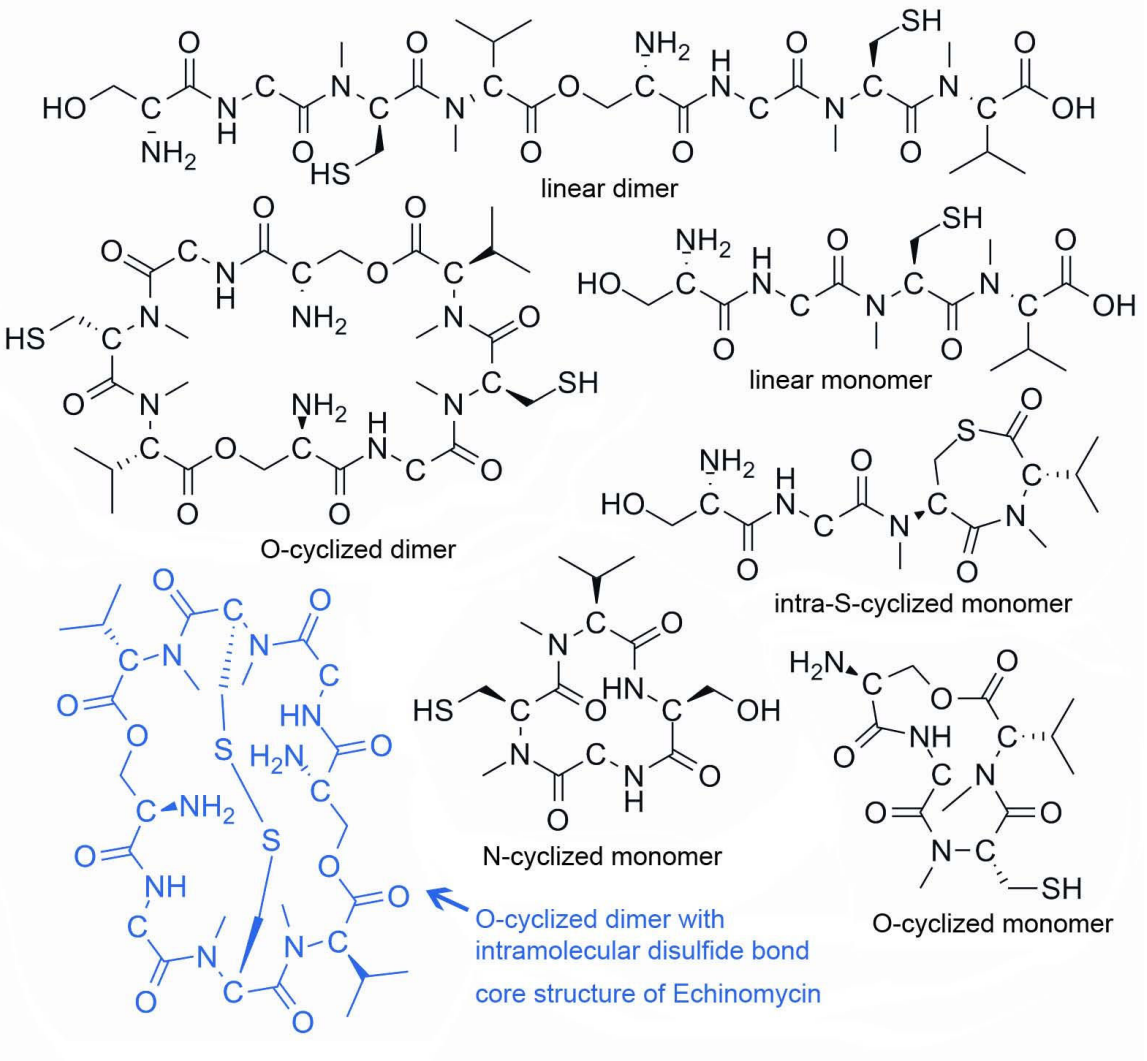
**Several predictions from the echinomycin cluster**. NP. searcher generated seven SMILES from the echinomycin cluster based on different cyclization modes and the presence or absence of dimerization and disulfide linkages. The highlighted blue molecule is the core of the actual structure.

**Figure 5:**
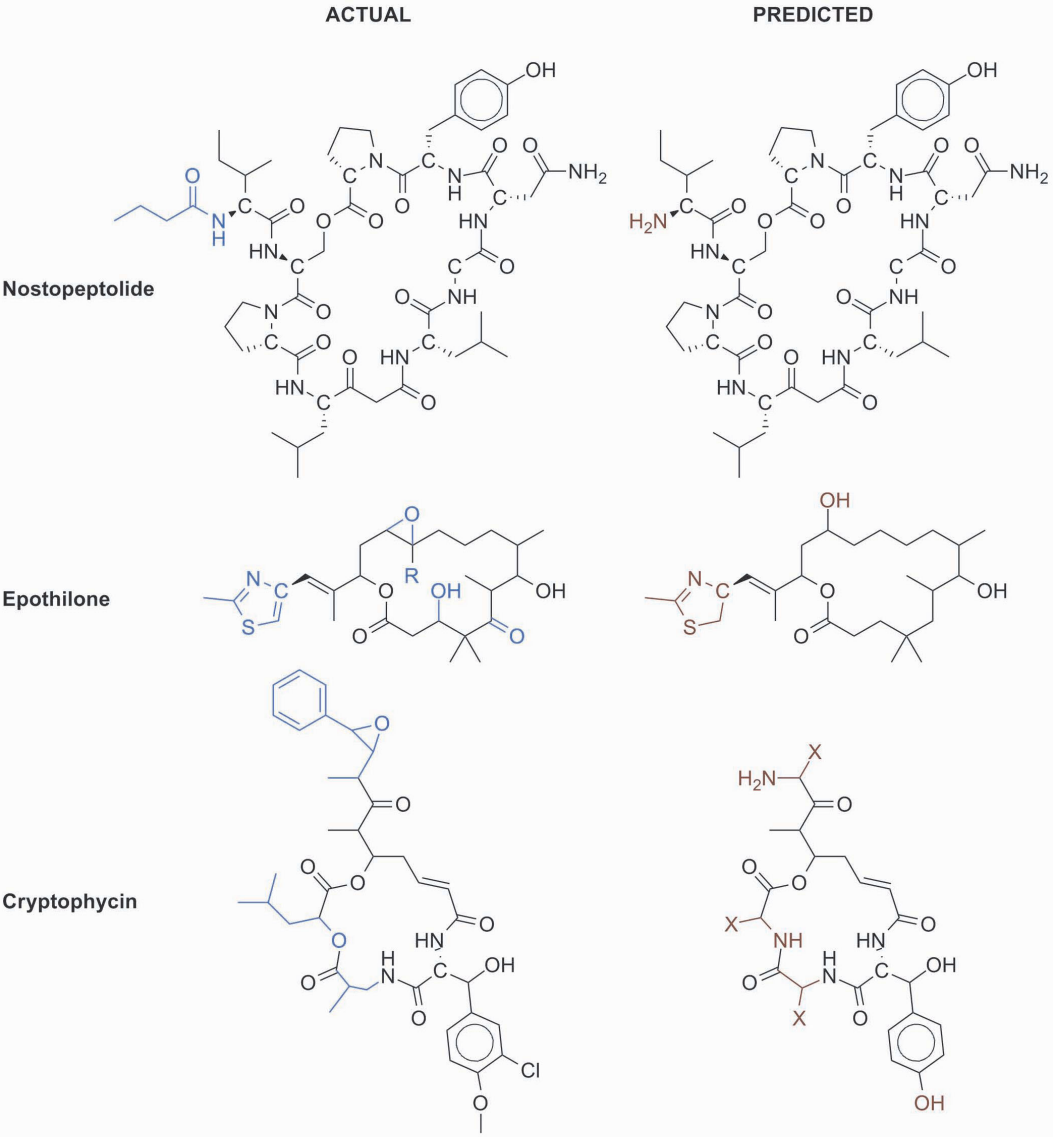
**Comparisons between predicted and actual structures**. The left column depicts the actual structures of molecules as verified chemically while the right column depicts the closest prediction from NP.searcher. Most unknown polyketide or peptide units, as seen in the cryptophycin example, result from novel signature sequences that do not match well with any specificity codes from our database of specificity codes compiled from literature sources. The actual chemical moieties are color-coded blue while the predicted or unrecognized moieties are shown in red.

We examined 79 natural product gene clusters from the databases NORINE and ASMPKS that produced metabolites with accessible structural information. Nearly 60% of PKS clusters and 80% of NRPS clusters were found to be co-linear and modular, meaning that the molecule could be constructed by transcription and translation of the nucleic acid sequences in one direction. The program was able to detect the twenty natural amino acids, twenty two unnatural amino acids, and other chain elongation intermediates in NRPS A domains and malonyl-CoA, methylmalonyl-CoA, ethylmalonyl-CoA, and methoxymalonyl-CoA AT specificity in PKSs. Various functional groups of amino acids and polyketide subunits contributed to the diversity of chemical structures, and specified for example, the dimerization of and disulfide bond formation within certain molecules such as echinomycin (Figure 4). They were also vital in forming the correct macrocyclized product through serine in nostopeptolide and leucic acid in cryptophycin (Figure 5).

Nucleophilic reactions such as heterocyclization hinted at formation of familiar groups such as a thiazole in epothilone [26,27] (Figure 5). Before performing heterocyclization reactions, there were seven structural predictions based on the biosynthetic gene cluster for this myxobacterial secondary metabolite. In three structural candidates the thiol of cysteine was either involved in dimerization or cyclization. In the other four candidate structures the sulfhydryl group was free to form a thiazole with the neighboring malonyl subunit. Performing heterocyclization on the molecules resulted in doubling of the four candidate metabolites to produce another four containing thiazole groups as opposed to malonyl and cysteinyl residues. After validating substrate specificity predictions on 254 adenylation domains from molecules in NORINE and 333 acyltransferase domains from polyketide synthases in ASMPKS, the accuracy of PKS acyltransferase substrate prediction was ~93% while NRPS adenylation substrate prediction was ~82% with ~77% of epimerization domains detected (Tables 1 and 2). Substrate specificity error was caused largely by unconventional starter units that did not possess conserved signature sequences stored in the database. We expect that this ambiguity will be ameliorated by future addition of new signature sequences to the database.

**Table 1:** Validation of PKS domain recognition

	**Co-linear/Non-co-linear Genes**	**AT^a^**	**KR^b^**	**DH^c^**	**ER^d^**
**Ratio**	23/39	309/333	291/293	159/177	59/60

**Percentage (%)**	59.0	92.8	99.3	89.8	98.3

^a ^AT, acyltransferase. ^b ^KR, ketoreductase. ^c ^DH, dehydrogenase. ^d ^ER, enoylreductase.

Highly unique PKS sequences that did not align with database-derived PKS AT, KR, DH, and ER sequences using BLAST resulted in some false negatives.

**Table 2:** Validation of NRPS domain recognition

	**Co-linear/Non-co-linear Genes**	**A^a^/Total # of A domains**	**E^b ^domains detected/Total # of E domains**
**Ratio**	32/40	208/254	30/39

**Percentage (%)**	80.0	81.9	76.9

^a ^A, adenylation. ^b ^E, epimerization.

Highly unique NRPS sequences that did not align with database-derived NRPS A and E sequences using BLAST resulted in some false negatives.

### Uncertainties in prediction

Of the co-linear clusters, there was an average of 5.19 predicted molecules based on the ability of various functional groups to enable macromolecular cyclization. The PK-focused ASMPKS database elicited an average of 5.84 predictions per cluster while those of the NRP-focused NORINE database had 4.64 predictions per cluster. This difference is caused by the greater number of hydroxyl groups on a PK that allow for cyclization. The core structure of the correctly predicted molecule differed in cases where detected domains were silent or inactive, or because of post-assembly tailoring modifications that were not incorporated by the program. Silent and inactive auxiliary PKS domains generated false positives, with 17% of DH, 12% of ER, and 6.8% of KR recognized even though they were inactive or silent based on the known structure of the molecule (Table 3). The best predictions aligned reasonably well with most of the actual structures (Figure 5). With greater understanding of the HMM inherent in the amino acid sequences that identify non-functional catalytic domains and those that define the presence and type of cyclization, our program will become more accurate, flexible and predictive.

**Table 3:** Validation of PKS false positives

	**KR^a^**	**DH^b^**	**ER^c^**
**Ratio**	20/293	30/177	7/60

**Percentage (%)**	6.8	16.9	11.7

^a ^KR, ketoreductase. ^b ^DH, dehydrogenase. ^c ^ER, enoylreductase.

Inactive or silent domains created a significant number of false positives in PKS pathway analysis.

Various modifications could be performed on the linear molecule predicted from the DNA sequence. NP.searcher immediately generates a macrocyclized version of the molecule upon determining the linear sequence. Oxygen, nitrogen, and sulfur methylation can be applied to the natural product pre- and post-assembly of the NRPs or PKs derived core structure. NP.searcher allows epimerization, carbon methylation, and three types of PKS processing (DH, ER, KR) only during assembly with halogenation, hydroxylation, heterocyclization, and glycosylation only during post-assembly modifications. Thus, there are eight assembly reactions, seven post-assembly tailoring reactions, and two termination strategies depending upon off-loading of a linear product or macrocyclization. Upon assembly and post-assembly modifications, NP.searcher outputs molecules as SMILES. Both simple and highly complex structures can be displayed through this text format, which is recognized by various software in common use such as ChemDraw, Daylight Depict, Molinspiration, Smile23d, InChI, and PubChem.

### Additional functions

In addition to assembling and predicting modular NRPS/PKS systems, the program is able to recognize trans-AT PKS and terpenoid gene clusters. The substrate sequence of trans-AT PKSs, unlike that of modular PKS derived AT domains is apparently determined by ketosynthases instead of acyltransferases [18]. Currently, the program can detect, though not build, a trans-AT PK molecule. This is accomplished by searching for consecutive KSs separated by less than 15 kilobases without intervening ATs. The program can also recognize terpenoid clusters by searching for essential genes of the mevalonate and non-mevalonate pathways: *ispH *and *mvd1*, respectively [28]. The innate ability of the program to perform post-assembly tailoring reactions allows users to enrich the database with additional core structure modifying enzymes. For example, incorporating cytochrome P450s and their corresponding hydroxylation and epoxidation reactions along with many other enzymes involved in natural product structure diversification is the next step for expanding the search engine's capabilities.

## Discussion

### The next frontier in drug discovery

In aiding investigators to discover secondary metabolites by structure prediction, this new natural products search engine enables drug discovery efforts to move one step closer to prediction of molecules for lead optimization. The development of SMILES analysis for biological activity coupled with the advancement of docking software that can accept SMILES as input and dock them with proteins opens the door to a useful new tool for pharmaceutical research and development. Building *in silico *models of newly discovered natural products to predict activity and perform docking is another incremental step beyond the trial-and-error method of drug development. The predicted natural products in SMILES format can be used to generate both 2D and 3D representations in assorted chemical software. 3D models produced from SMILES can be used for docking with proteins using well-established methods [29].

### Challenges to prediction

Currently, the best predicted structure of the molecule differs significantly in many cases from the actual molecule because of non-functional domains and unrecognized post-tailoring modifications. Thus, small molecule-target docking of the best predicted structure may not be representative of the actual molecular interaction. However, this is expected to improve with the deciphering of the determinants of inactive or silent auxiliary domains, along with further understanding of signature residues that determine the absence or presence of cyclization, the nature of cyclization, the type of post-assembly tailoring modifications, and their sites of action.

Important limitations remain in this initial version of NP.searcher and represent challenges for the future in this open source tool. For example, an incomplete ability to predict rare substrates because of a limited database of known natural product starter and extender units can lead to false or deficient predictions. However, finding compounds with unknown groups during genome mining might motivate the pursuit of predicted molecules as is the case with cryptophycin. These predictions were made from only a small number of known sequences and signatures stored by NP.searcher. Adding the signature sequences of these unique substrates to the search engine would reduce significantly the number of unknown subunits and increase program performance.

### New sources for mining

The emerging views from marine invertebrate (e.g. sponges, tunicates, ascidians) and terrestrial microbial symbionts reveals that trans-AT PKS systems specify synthesis of a large proportion of novel natural products, and thus it is crucial to be able to genome-mine potential products from these non-traditional clusters [18]. Furthermore, plant genomes should reveal a cornucopia of unexplored and diverse NRPs, PKs, and terpenoids that have potential therapeutic applications [30]. With the development of heterologous expression of plant secondary metabolite pathways and better understanding of plant natural product metabolic systems, these organisms should increasingly become an attractive source of valuable compounds [31,32]. Though terpenoid biosynthetic mechanisms are yet to be elucidated as well as that of NRPSs and PKSs, the abundance of medicines that may be produced from them and the scientific curiosity in plant biosynthesis might drive both industry and academia to tackle terpenoid pathways more aggressively in the future [33].

### The keys to automated elucidation

With genome sequencing rapidly becoming more affordable, the bottleneck becomes the ever-elusive ability to predict small molecule structure, and more challengingly, protein three-dimensional structures from two-dimensional specifications. Although, there are sure to be an enormous number of novel enzymes to discover and characterize functionally, the existing database of known proteins and increasingly refined biochemical tools such as metabolic and gene expression profiling coupled with heterologous protein expression will accelerate solving enzymatic puzzles [34]. Enzymes with low substrate specificity may be difficult to analyze using hidden Markov models and the time to use them *in silico *may not come until we can accurately simulate protein dynamics.

With better understanding of how natural products are genetically and enzymatically determined and the advance of rapid genomic scanning technologies, there is a need to extract chemical knowledge from genetic information more efficiently for potential applications. Taking advantage of the recent advances in natural products domain recognition, NP.searcher decodes natural product gene clusters into molecules and brings to the forefront the ability to recognize thousands of new secondary metabolites. On the more basic level, this program can function as an editing device to compose natural product molecules. With the development of greater protein engineering capabilities, this program will enable biologists and chemists to envision possible NRPs and PKs to design de novo pathways by metabolic engineering or synthetic biology approaches. At the most advanced and useful level, NP.searcher may read through thousands of gene clusters and automatically construct and screen potential natural product molecules from large databases of uncharacterized microbial genome or mixed metagenome sequences. In another future application, the development of better protein three-dimensional modeling, the program will seek to employ reverse engineering to provide DNA sequences required to prescribe the biosynthesis of a particular natural product molecule.

### A natural products search engine

In addition to extensively searching through chemical space, NP.searcher performed intramolecular reactions in output structures as seen with echinomycin. Such dynamic *in silico *enzymology will be necessary to elucidate compounds with the complexity of the NRPS-derived antibiotic vancomycin [35]. Given the diverse chemical arsenal found in Nature, such an extensive search capability promises to uncover interesting candidate metabolites from NP.searcher by performing various intramolecular reactions on a natural product molecule. Moreover, the SMILES output of the program can be applied to other software to analyze and predict biological activities relevant to selected drug targets. In addition to the challenges of predicting bioactivity from SMILES, resulting 2D and 3D structures, and predicting cytotoxicity and drug action for a predicted structure, other important challenges remain to be addressed such as elucidation of terpene biosynthesis and non-co-linear and dispersed synthetase systems in microbes and plants. Accordingly, the broad potential of cost-efficient genome sequencing coupled to rapid and accurate prediction of secondary metabolic products and biological activity provides an urgent motivation to accomplish these objectives more quickly and effectively.

## Conclusion

NP.searcher was developed to scan rapidly microbial genomes for secondary metabolite biosynthetic gene clusters, and output candidate nonribosomal peptide and polyketide natural products in SMILES format, enabling immediate decoding of DNA to produce 2D and 3D structures in widely available software. The ability to recognize novel NRP and PK products will grow with continuous updating of the search engine's database of adenylation and acyltransferase signature sequences for various amino acid and polyketide starter and extender units. The value of NP.searcher is likely to improve with addition of algorithms built on further proteomic analysis that reveal the basis for post-assembly tailoring steps such as cyclization, glycosylation, methylation, and various other common reactions, further enhancing the output of structural predictions. With the development of faster and cheaper genome sequencing technologies, NP.searcher may be increasingly useful in the rapid screening of suitable natural product drug candidates directly from genomic information.

## Availability and Requirements

Project name: Natural products search engine

Project home page: 

Login: temp; Password: temp

Operating system: Linux and web-based

Programming language: C++

Any restrictions to use by non-academics: no, open-source

## Abbreviations

PKS: polyketide synthase; AT: acyltransferase; DH: dehydratase; ER: enoylreductase; KR: ketoreductase; NRPS: nonribosomal peptide synthetase; A: adenylation; E: Epimerization; SMILES: simplified molecular input line entry specification; HMM: hidden Markov model.

## Authors' contributions

HTL and DHS conceived the program. HTL performed most of the programming and validation, and drafted the manuscript. PMG partnered with HTL in writing the program in the beginning stages, conceived of using BLAST to identify similar signature sequences from the database, and assisted in revising the manuscript. JZ built the website to run the program online. SGT provided initial guidance and support for working on the program and assisted in revising the manuscript. DHS provided initial and continued support and oversight for the program and helped draft the manuscript. All authors read and approved the final manuscript.
